# Cross-order host switches of hepatitis C-related viruses illustrated by a novel hepacivirus from sloths

**DOI:** 10.1093/ve/veaa033

**Published:** 2020-04-25

**Authors:** Andres Moreira-Soto, Francisco Arroyo-Murillo, Anna-Lena Sander, Andrea Rasche, Victor Corman, Birthe Tegtmeyer, Eike Steinmann, Eugenia Corrales-Aguilar, Nicolas Wieseke, Judy Avey-Arroyo, Jan Felix Drexler

**Affiliations:** Charité-Universitätsmedizin Berlin, corporate member of Freie Universität Berlin, Humboldt-Universität zu Berlin, and Berlin Institute of Health, Institute of Virology, Berlin 10117, Germany; Virology-CIET, Faculty of Microbiology, University of Costa Rica, San José, Costa Rica; The Sloth Sanctuary of Costa Rica, Limon, Costa Rica; Charité-Universitätsmedizin Berlin, corporate member of Freie Universität Berlin, Humboldt-Universität zu Berlin, and Berlin Institute of Health, Institute of Virology, Berlin 10117, Germany; Charité-Universitätsmedizin Berlin, corporate member of Freie Universität Berlin, Humboldt-Universität zu Berlin, and Berlin Institute of Health, Institute of Virology, Berlin 10117, Germany; Charité-Universitätsmedizin Berlin, corporate member of Freie Universität Berlin, Humboldt-Universität zu Berlin, and Berlin Institute of Health, Institute of Virology, Berlin 10117, Germany; Institute for Experimental Virology, TWINCORE Centre for Experimental and Clinical Infection Research, a Joint Venture Between the Medical School Hannover (MHH) and the Helmholtz Centre for Infection Research (HZI), Hannover 30625, Germany; Department of Molecular and Medical Virology, Faculty of Medicine, Ruhr-University Bochum, Bochum 44801, Germany; Virology-CIET, Faculty of Microbiology, University of Costa Rica, San José, Costa Rica; Swarm Intelligence and Complex Systems Group, Department of Computer Science, Leipzig University, Leipzig, Germany; The Sloth Sanctuary of Costa Rica, Limon, Costa Rica; Charité-Universitätsmedizin Berlin, corporate member of Freie Universität Berlin, Humboldt-Universität zu Berlin, and Berlin Institute of Health, Institute of Virology, Berlin 10117, Germany; German Centre for Infection Research (DZIF), Germany

**Keywords:** sloth, hepatitis C virus, Costa Rica, evolution, host switch

## Abstract

The genealogy of the hepatitis C virus (HCV) and the genus *Hepacivirus* remains elusive despite numerous recently discovered animal hepaciviruses (HVs). Viruses from evolutionarily ancient mammals might elucidate the HV macro-evolutionary patterns. Here, we investigated sixty-seven two-toed and nine three-toed sloths from Costa Rica for HVs using molecular and serological tools. A novel sloth HV was detected by reverse transcription polymerase chain reaction (RT-PCR) in three-toed sloths (2/9, 22.2%; 95% confidence interval (CI), 5.3–55.7). Genomic characterization revealed typical HV features including overall polyprotein gene structure, a type 4 internal ribosomal entry site in the viral 5′-genome terminus, an A–U-rich region and X-tail structure in the viral 3′-genome terminus. Different from other animal HVs, HV seropositivity in two-toed sloths was low at 4.5 per cent (3/67; CI, 1.0–12.9), whereas the RT-PCR-positive three-toed sloths were seronegative. Limited cross-reactivity of the serological assay implied exposure of seropositive two-toed sloths to HVs of unknown origin and recent infections in RT-PCR-positive animals preceding seroconversion. Recent infections were consistent with only 9 nucleotide exchanges between the two sloth HVs, located predominantly within the E1/E2 encoding regions. Translated sequence distances of NS3 and NS5 proteins and host comparisons suggested that the sloth HV represents a novel HV species. Event- and sequence distance-based reconciliations of phylogenies of HVs and of their hosts revealed complex macro-evolutionary patterns, including both long-term evolutionary associations and host switches, most strikingly from rodents into sloths. Ancestral state reconstructions corroborated rodents as predominant sources of HV host switches during the genealogy of extant HVs. Sequence distance comparisons, partial conservation of critical amino acid residues associated with HV entry and selection pressure signatures of host genes encoding entry and antiviral protein orthologs were consistent with HV host switches between genetically divergent mammals, including the projected host switch from rodents into sloths. Structural comparison of HCV and sloth HV E2 proteins suggested conserved modes of hepaciviral entry. Our data corroborate complex macro-evolutionary patterns shaping the genus *Hepacivirus*, highlight that host switches are possible across highly diverse host taxa, and elucidate a prominent role of rodent hosts during the *Hepacivirus* genealogy.

## 1. Introduction

The hepatitis C virus (HCV; genus *Hepacivirus*, family *Flaviviridae*) infects millions of people worldwide, leading to thousands of deaths annually (Lavanchy 2011). For many years, HCV and a distantly related virus of likely non-human primate origin termed GBV-B were the only known hepaciviruses (HVs) (Simons et al. 1995). During recent years, diverse HVs have been discovered from five different mammalian host orders, namely Chiroptera (different bat genera and species), Perissodactyla (odd-toed ungulates, horses, and donkeys), Artiodactyla (even-toed ungulates, cattle), Primates (humans, colobus monkeys, and tamarins), and Rodentia (different rodent genera and species; summarized in Rasche et al. (2019)). These orders all belong to a mammalian clade termed Boreoeutheria that arose approximately 87 million years ago (mya) (Foley et al. 2016).

Despite the newly discovered animal homologs of HCV, the evolutionary origins of HCV and macro-evolutionary patterns shaping the genealogy of the genus *Hepacivirus* remain unresolved. On the one hand, rodent- and bat-associated HVs are genetically highly diversified, which suggests that bat and rodent hosts may have played an important role during the genealogy of extant HVs. This hypothesis is reminiscent of the evolutionary origins of other human hepatitis viruses in ancestors carried by small mammals and the important role of those animals for virus evolution in general (Drexler et al. 2013, 2015; Quan et al. 2013; Olival et al. 2017; Rasche et al. 2019). On the other hand, HCV is highly diversified in humans and HVs found in hosts other than rodents and bats are also genetically highly divergent, for example, cattle HVs (Simmonds 2013; Corman et al. 2015). HVs are hardly transmissible to heterologous hosts experimentally (summarized in Rasche et al. (2019)). Nonetheless, the majority of human hepatitis viruses likely evolved through ancestral host switches from yet unknown reservoirs (summarized in Rasche et al. (2019)). Host switches also must have occurred during the genealogy of extant HVs, exemplified by the relatively close phylogenetic relatedness of HCV and equine HVs, which is in contrast with the phylogenetic distance between their equid and primate hosts (Pybus and Gray 2013; Walter et al. 2017).

**Figure 1.**
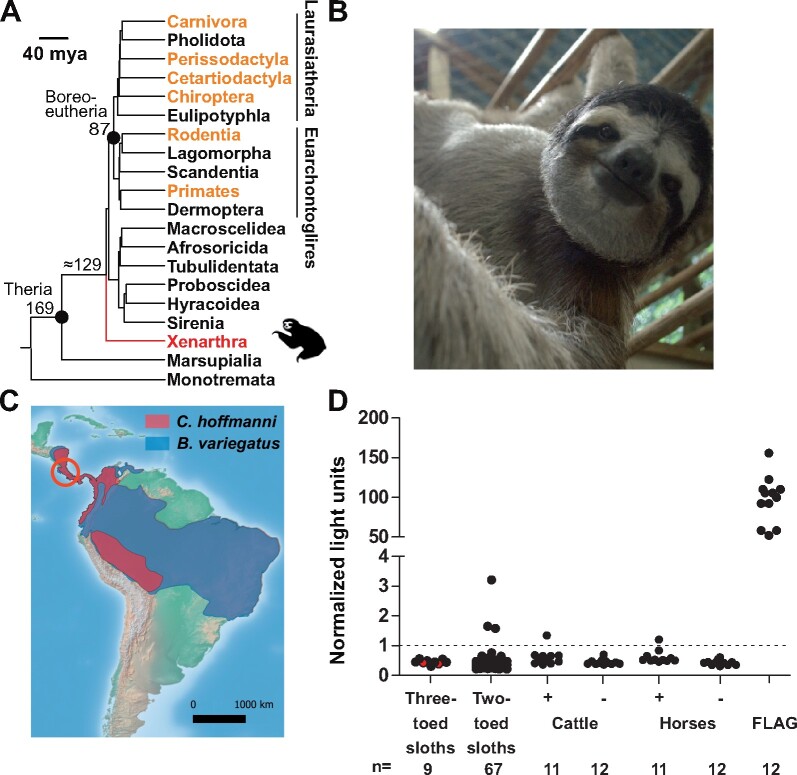
Phylogeny and hepacivirus serology of sloths. (A) Mammalian phylogeny showing the time of divergence between Xenarthra and therians, according to Foley et al. (2016). Mya: million years ago; known hepacivirus host orders are depicted in orange. (B) Hepacivirus-positive three-toed sloth from this study. Photo: Andres Moreira-Soto. (C) Distribution of *B.variegatus* in blue and *C.hoffmanni* in red according to the IUCN Red List (https://www.iucnredlist.org/), mapped using QGIS (www.qgis.org) and open source data freely available from Natural Earth (http://www.naturalearthdata.com). Costa Rica is encircled in red. (D) Luciferase immunoprecipitation system (LIPS) assay; black dots correspond to the normalized light units. RT-PCR-positive three-toed sloths are depicted in red. Hepacivirus seropositive (+) and seronegative (−) cattle and horses were used to assess LIPS assay cross-reactivity. Dotted line denotes the normalized cutoff. Anti-FLAG, expression controls.

We have shown previously for a novel marsupial hepatitis A virus (HAV) that hepatitis viruses from outlier mammals can inform analyses of viral macro-evolutionary patterns (de Oliveira Carneiro et al. 2018). The clade Xenarthra comprises genetically diverse mammals such as armadillos, anteaters, and sloths that diverged from other placental mammals approximately 100 mya (Delsuc et al. 2001; Murphy et al. 2001) (Fig. 1A). Extant species of sloths belong to the *Bradypus* and *Choloepus* genera, that diverged more than 30 mya (Presslee et al. 2019). Sloths have mainly been investigated for arthropod-borne viruses by serological tools (Seymour et al. 1983a,b), and genomic data on viruses infecting sloths is scarce compared to other mammalian hosts (Medlin et al. 2016; Catenacci et al. 2018; de Oliveira Filho et al. 2020). Here, we describe novel HVs in sloths using molecular, serologic, and evolutionary tools to reconstruct the genealogy of the genus *Hepacivirus*.

## 2. Materials and methods

### 2.1 Fieldwork

Sampling of sloths in captivity was carried out in the Sloth Sanctuary, Penshurt, Limón, Costa Rica during 2014. The sloths in the sanctuary correspond to the two extant sloth species in Costa Rica: two-toed sloths (*Choloepus hoffmanni*) and three-toed sloths (Fig. 1B, *Bradypus variegatus*).The protocol and procedure for sampling was approved by the National Council in the Management of Biodiversity (resolution R-026-OT-CONAGEBIO) according to international animal health standards. Briefly, sloths were anesthetized by trained veterinarians for the purpose of teeth and nail grooming and blood from the jugular vein and an anal swab were collected.

### 2.2 Serology

To assess HV seroprevalence in the sloth population, we developed a luciferase immunoprecipitation system (LIPS) assay as described previously (Burbelo et al. 2009). Briefly, Cos-1 cells were transfected with the pREN2 expression vector that contained the NS3/NS4A coding region from the sloth HV and a C-terminal FLAG-tag downstream of a Renilla luciferase coding region. Crude protein extracts were obtained as described previously (Burbelo et al. 2009) for use as antigen. Serum samples (1 μl) were mixed with 49 μl of buffer A, incubated for 1 h and 10^7^ relative light units (RLU) of transfected Cos-1 cell extract in buffer A were added to each well. The plate was incubated at 4 °C overnight. Next, a suspension of Ultralink protein A/G beads (Pierce Biotechnology, USA) was added and incubated at room temperature for 1 h. After repeated washing, RLU were measured. The cutoff was derived from the mean value of six wells containing only buffer A, the Renilla-NS3/NS4A protein and A/G beads as described previously plus ten standard deviations (Burbelo et al. 2012). Sample-derived RLU were normalized by dividing through plate-specific cutoff values. To analyze cross-reactivity of antibodies elicited by divergent HVs, we tested seropositive and seronegative horse-derived sera according to an equine HV NS3/4A-based LIPS assay (Reichert et al. 2017) and seropositive and seronegative cattle-derived sera according to a cattle HV NS3/4A-based LIPS assay with the sloth HV NS3/4A-based LIPS assay. Each LIPS run contained control reactions consisting of 1 µl of a mouse monoclonal anti-FLAG antibody (1 mg/ml; Sigma-Aldrich, USA).

### 2.3 Molecular analyses

Viral RNA from the different samples was extracted using the MagNA Pure 96 Viral NA Small Volume Kit (Roche Molecular Systems, USA). Screening for HVs was done using a sensitive nested reverse transcription polymerase chain reaction (RT-PCR) assay targeting conserved regions as described previously (Drexler et al. 2013). For complete genome sequencing, next-generation sequencing (NGS) was performed in pooled samples of the positive sloths. Library construction and Illumina MiSeq sequencing was carried out by using the SuperScript^®^ One-Cycle cDNA Kit, the Nextera XT DNA Library Preparation Kit and V3 chemistry (2 × 300 bases read length) according to the manufacturers’ instructions. 1,875,042 million high-quality sequence reads were obtained from the library, merged by using FLASH and mapped against the screening RT-PCR fragment in Geneious 9.1.8. After performing multiple iterations, a total of 25,964 reads could be assembled to one complete consensus genome. After the full genome was assembled, confirmatory long-range PCRs assays were performed as previously described on the two individual positive samples (Drexler et al. 2013). Briefly, nested- or hemi-nested-assays covering approximately 1,000 base pairs each were designed based on the NGS data and Sanger sequenced (Supplementary Table S1). Genome termini were confirmed using a rapid amplification of cDNA ends (RACE) strategy (Roche, Penzberg, Germany). Quantification of viral loads was done using a strain-specific real-time RT-PCR, using in vitro transcribed RNA controls as described previously (Supplementary Table S1) (Drexler et al. 2009).

### 2.4 In silico analyses

Genome annotations were done using Geneious 9.1.8 in analogy to known HVs (Kearse et al. 2012). Translated sequences were aligned using MAFFT (Katoh 2002). Recombination analyses were carried out using RDP4 (Martin et al. 2015). A possible recombination event was considered only when three or more algorithms were positive, all other potential recombination events were discarded. Sequence distances were calculated using a sliding window of 400 and a step size of 200 nt and mean minimum folding energy differences (MFED) were calculated using a sliding window of 250 and a step size of 30 nt in SSE V1.3 (Simmonds 2012). Signal peptidase cleavage sites were predicted using Geneious 9.1.8 (Kearse et al. 2012) and SignalP 4.1 (Petersen et al. 2011). N-glycosylation sites were predicted using NetNGlyc 4.0 (Steentoft et al. 2013). Secondary structure predictions were done using Mfold (Zuker 2003). HV entry receptor and mitochondrial antiviral signaling protein (MAVS) sequences were retrieved from GenBank, pairwise distance was calculated in MEGA X (Kumar et al. 2018). Site-specific selection pressure analyses were done using Single Bayesian Approximation (FUBAR) (Murrell et al. 2013), Fixed Effect Likelihood (FEL), Mixed Effects Model of Evolution (MEME), Single Likelihood Ancestor Counting (SLAC), and Random Effect Likelihood (REL) and gene-wide selection pressure analyses were done using Branch-site Unrestricted Statistical Test for Episodic Diversification (BUSTED), all implemented within the HyPhy package via the Datamonkey platform (Weaver et al. 2018) and using the HKY85 substitution model in all cases. In addition, codon-based models (CodeML program) implemented in the PamLX 1.3.1 software package using the codon frequency model F61 (Xu and Yang 2013) were used to test for positive selection in individual codons. Site model M7 (beta) that only allows codons to evolve neutrally or under purifying selection was compared to M8 (beta & ω) which allows codons to evolve under positive selection using likelihood ratio tests in the PamLX package (Xu and Yang 2013). Thermodynamic modeling of sloth HV E2 was done on the HCV crystal structure (Lavie et al. 2015) by use of Chimera and ESPript 3.0 (Pettersen et al. 2004; Robert and Gouet 2014).

### 2.5 Phylogenetics

Bayesian phylogenies were generated using MrBayes V3.1 using a WAG amino acid substitution model (Whelan and Goldman 2001; Ronquist et al. 2012). Trees were run for two million generations, sampled every hundred steps. After an exclusion of 5,000 of the total 20,000 trees as burn-in, final trees were annotated with TreeAnnotator and visualized with FigTree from the BEAST 1.10 package (Suchard et al. 2018). For co-evolutionary analyses, HV and host cytochrome B sequences were obtained from GenBank and used for reconstruction of phylogenetic relationships as described above. Nexus input files for co-evolutionary analyses were generated in MEGA X (Kumar et al. 2018). Several taxonomic constrains were used for accuracy of host phylogenies according to established species trees as previously described (Drexler et al. 2015). ParaFit (Legendre et al. 2002) was run in R (V3.4.1), through the Rstudio environment (V1.0.153), with the packages APE (V4.1), and Vegan (V2.4-3) and 100,000 random permutations of virus–host associations with test for statistical significance. Individual host–virus associations were evaluated using the ParaFitLink1 statistical test (Legendre et al. 2002). CoRe-PA (Merkle et al. 2010) was used to reconstruct evolutionary histories considering four distinct evolutionary events (co-speciation, sorting, duplication, and host switch). The most parsimonious costs per event were determined automatically during 5,000 cycles. Host–virus associations were randomized to yield hundred replicates as previously described (de Oliveira Carneiro et al. 2018). Host–virus associations were constructed using an automated cost model (abbreviated *a*), the same automated model but excluding reconstructions without at least one host switch (abbreviated *w*/*s*), and reconstructions maximizing co-speciations by using negative co-speciation costs (abbreviated *co*). Ancestral state reconstructions (ASRs) were done in a Bayesian framework using BEAST 1.10 (Suchard et al. 2018) as described previously (de Carvalho Dominguez Souza et al. 2018). ASRs in a parsimony-based framework were performed using Mesquite (Maddison and Maddison 2009) as described previously (Drexler et al. 2012).

## 3. Results

### 3.1 Sampling

During August and September 2014, we collected seventy-six blood samples (sixty-seven from two-toed sloths and nine from three-toed sloths) and corresponding anal swabs from individual sloths in captivity (‘Sloth Sanctuary’, www.slothsanctuary.com) in Limón, Costa Rica. The conservation status of these sloth species is listed as least concern in the IUCN red list, but their population is declining due to continuous deforestation (Superina et al. 2010). Both sloth species are distributed from Central to South America (Fig. 1C).

### 3.2 Hepacivirus screening

Two out of nine sera from three-toed sloths (Fig. 1B and Table 1) were positive in the screening RT-PCR (Drexler et al. 2013), corresponding to a 22.2 per cent detection rate (2/9; adjusted Wald 95% confidence intervals (CIs), 5.3–55.7). No two-toed sloth was RT-PCR-positive. Viral loads were high at 4.7 × 10^6^ and 2.2 × 10^8^ RNA copies/ml serum (Table 1). In contrast, no fecal swab was positive, including swabs from those two animals that yielded positive results in serum. None of the two RT-PCR-positive animals showed signs of clinical disease. Measurement of liver enzymes to evaluate the degree of potential sloth HV-mediated liver damage was not possible due to insufficient sample volumes. Notably, sloths differ from other mammals in their unusually low body temperature and metabolic rates that are associated with their toxic diet (Cliffe et al. 2018). It cannot be excluded that these animals may be differentially affected by systemic viral infections such as those potentially caused by HVs, compared to other mammals (de Oliveira Filho et al. 2020). Hepaciviral infection dynamics and symptoms in sloths thus require further investigations including relatively large numbers of animals, because liver enzymes remain in the normal range for long periods in about 30 per cent of humans infected with HCV, and were not consistently elevated in animals infected with equine HV, cattle HV, and Norway rat HV (Calvaruso and Craxì 2009; Baechlein et al. 2015; Walter et al. 2017; Trivedi et al. 2018).

**Table 1.** Molecular and serological findings in hepacivirus-infected sloths.

ID	Sex	Species	Age	RT-PCR (copies/ml)	LIPS (NLU)
B31	M	*B.variegatus*	Adult	+ (4.7 × 10^6^)	−
B32	M	*B.variegatus*	Adult	+ (2.2 × 10^8^)	−
C141	F	*C.hoffmanni*	Adult	−	+ (3.24)
C72	M	*C.hoffmanni*	Adult	−	+ (1.68)
C66	F	*C.hoffmanni*	Adult	−	+ (1.61)

Cutoff for negative samples: 1 NLU.

ID, identification; LIPS, luciferase immunoprecipitation assay; NLU, normalized light units; PCR, polymerase chain reaction.

Challenges of seroprevalence studies in sloths include the lack of standardized methodology. To test for antibodies against sloth HV, a LIPS assay was established. Using the sloth HV-specific LIPS assay, HV seroprevalence in two-toed sloths was low at 4.5 per cent (3/67; CI, 1.0–12.9) whereas all three-toed sloths were seronegative, including the two RT-PCR-positive animals (Table 1) (Fig. 1D). The lack of detectable antibodies in the two RT-PCR-positive sloths was surprising. Sloth immune responses are poorly understood, but anecdotal evidence of experimental infections with Saint Louis encephalitis virus (SLEV) suggests delayed mounting of antibody responses compared to other mammals (Seymour et al. 1983a). SLEV belongs to the genus *Flavivirus*, which alike the genus *Hepacivirus* belongs to the viral family *Flaviviridae*. Relatively delayed seroconversion may thus be transferable to sloth HV infections. On the one hand, lack of detectable antibodies and close genetic relatedness of the HV genomes derived from the two sloths is thus consistent with acute infections of the RT-PCR-positive three-toed sloths preceding seroconversion. On the other hand, persistent HV infections may down-regulate antibody responses to undetectable levels in serologic tests, such as documented for HCV-infected individuals (Vanhommerig et al. 2014) and for the genetically related human pegiviruses (Coller et al. 2020). We thus cannot exclude that at least one of the sloths may be persistently infected with the sloth HV.

During prior studies, we described reactivity of rodent and bat sera with HCV antigens, suggesting that antibodies elicited by distinct HVs may cross-react in serological assays (Drexler et al. 2013). To assess specificity of our LIPS assay, we tested sera from cattle and horses with known reactivity against homologous equine and cattle HV antigens. Using the sloth HV NS3/4A-based LIPS assay with those sera, two out of twenty-two (9.1%; CI: 1.3–29.0) cattle- and horse-derived sera that were seropositive in tests relying on homologous cattle or equine HV antigens also showed reactivity above the cutoff, with similar normalized light units to two out of three sloth sera considered seropositive (Fig. 1D) (Table 1). None of twelve seronegative cattle-derived sera and none of twelve seronegative horse-derived sera showed reactivity above the cutoff in the sloth HV LIPS assay. Combining the serologic data from all sera derived from sloths, cattle and horses, the sloth HV LIPS assay showed a specificity of 98.3 per cent (CI: 94.1–99.5) and a negative predictive value of 100.0 per cent (CI: 96.8.6–100.0). This suggested limited cross-reactivity in NS3/4A-based LIPS assays and underlines that the observed antibody responses in two-toed sloths were not necessarily elicited by the HV described from three-toed sloths.

### 3.3 Species demarcation of the sloth HV

**Figure 2.**
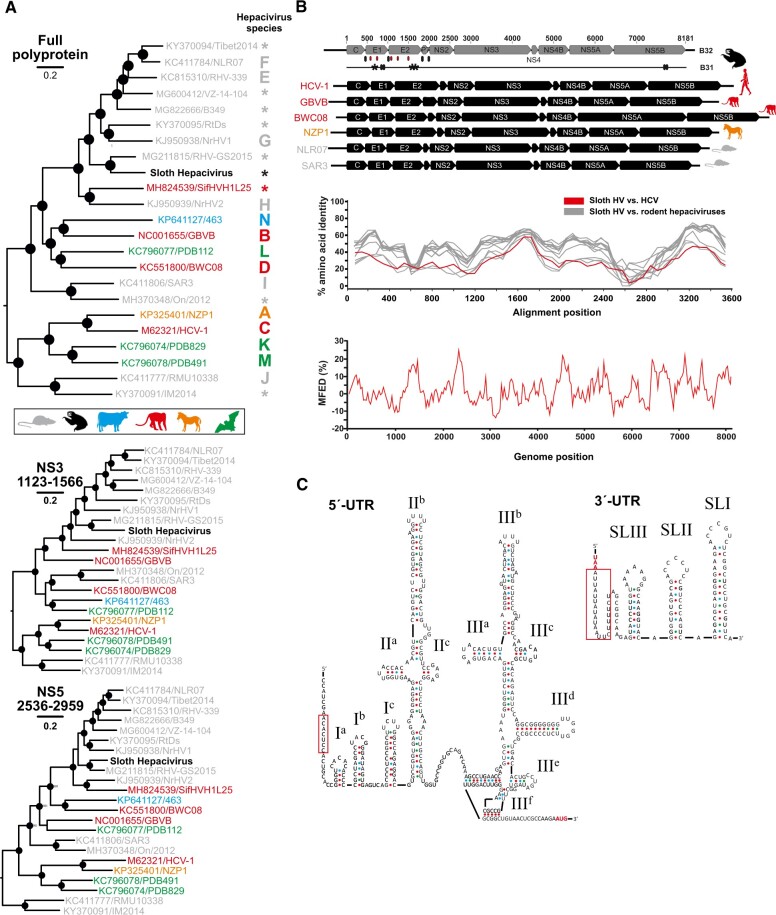
Genomic characterization of the sloth hepacivirus. (A) Bayesian phylogeny of the complete hepacivirus translated polyprotein gene. Bottom, Bayesian phylogeny of the NS3 and NS5B fragments used for species demarcation criteria according to Smith et al. (2016). The branch to Wenling shark virus, GenBank accession number: KR902729 used as an outgroup was truncated for graphical reasons. Filled circles correspond to posterior probability support of grouping above 0.9. Trees were constructed with recognized species and unclassified viruses for which the full polyprotein was available. Asterisks denote unclassified hepaciviruses. (B) Full genome organization of both sloth HV strains (B31 and B32) compared to other HV species. Typical hepaciviral proteins are shown in gray boxes for sloth HV and in black for selected HV species. Black diamonds on the bottom indicate predicted signal peptidase cleavage sites; red diamonds indicate N-linked glycosylation sites. Synonymous mutations in sloth HV strain B31 are shown as black lines, and non-synonymous mutations as asterisks. Bottom, comparison of amino acid sequence identity of sloth HV versus HCV (shown in red), and versus rodent hepaciviruses (shown in gray; refer to panel A for GenBank accession numbers). Below, mean minimum folding energy differences (MFED) of sloth HV. (C) IRES prediction with stem-loops numbered next to structures. The microRNA122-binding site is encased in red. Right, 3′-end secondary structure of sloth HV with the A–U-rich region encased in red.

In a Bayesian phylogeny relying on the full polyprotein gene, the sloth HV clustered within a relatively large clade composed exclusively by rodent HVs, except a recently described lemur HV from Madagascar (Fig. 2A, top) (Canuti et al. 2019). The sloth HV formed a phylogenetically discernible subcluster together with a rodent HV termed MG211815/RHV-GS2015 derived from a Chinese Daurian ground squirrel (*Spermophilus dauricus*) (Li et al. 2019) and two distinct HVs obtained from Norway rats (*Rattus norvegicus*) in the USA (Firth et al. 2014). The highest sequence identity between the sloth HV and the genetically most similar rodent viruses was observed in the NS3 and NS5B proteins (Fig. 2B). Defined segments of these two HV proteins have been suggested as species demarcation criteria, using a <0.25 NS3 and <0.3 NS5B amino acid sequence distance threshold to define a new HV species (Smith et al. 2016). Translated sequence distances of the sloth HV compared to defined HV species and to unclassified rodent HVs ranged from 0.32 to 0.55 in NS3 and 0.23 to 0.58 in NS5B segments. Consistent with phylogenetic reconstructions, the lowest amino acid sequence distances (0.32 in NS3 and 0.23 in NS5B) were observed in comparison to the unclassified rodent HV from China (Li et al. 2019). According to the NS5B threshold, but not the NS3 threshold, both the Chinese rodent and the Costa Rican sloth HVs would be conspecific. Although recombination between different HV species may be possible (Theze et al. 2015), recombination was neither reliably supported in formal analyses using the sloth HV complete polyprotein gene sequence, nor in the phylogenetic trees reconstructed using the NS3 and NS5B segments used for distance-based species demarcation (Fig. 2A, middle and bottom), refuting recombination as an explanation of the discrepant NS3 and NS5B sequence distance comparisons. Similar taxonomic problems have been documented in other rodent HVs, and a host demarcation criterion was used to separate these viruses into distinct HV species (Smith et al. 2016). The sloth HV may thus represent a new HV species.

### 3.4 Genomic characterization of the sloth HV

The full sloth HV polyprotein gene comprised 8,181 nucleotides (nt), similar to the length of previously characterized rodent HVs and shorter than primate and equine HVs (Fig. 2B). The sloth HV nonetheless contained a typical HV structure and sequence, encoding the predicted structural Core, E1 and E2 proteins and the non-structural proteins p7, NS2, NS3, NS4A, NS4B, NS5A, and NS5B (Fig. 2B). Interestingly, the two sloth HV strains showed only nine nt substitutions between each other. Seven of those substitutions occurred in the E1/E2 proteins (C268A, G276C, G279C in E1 and A1171U, U1172A, A1173U, G1189A in E2; Fig. 2B) and two in the NS5B protein (U7245A, A7251U; Fig. 2B) that translated into three amino acid substitutions in the E1/E2 proteins (L89I in E1 and I390Y, A396T in E2; asterisks in Fig. 2B). The accumulation of substitutions in the E1/E2 proteins was reminiscent of relatively higher variability of these regions within HCV (Gray et al. 2011), likely associated with micro-evolutionary processes associated with host immune evasion (Forni et al. 2018). The HCV evolutionary rate has been estimated at about 1.5 × 10^−3^ substitutions/site/year (Gray et al. 2011). If a similar evolutionary rate applied for sloth HVs, the low number of substitutions between the two sloth HV strains suggests a recent common origin of those viruses. The putative sloth HV E1 and E2 proteins contained several predicted N-glycosylation sites (two and three, respectively; shown in red (Fig. 2B)) and predicted signal peptidase cleavage sites (shown in black (Fig. 2B)) as observed for other HVs (Corman et al. 2015). A high number of genome-scale ordered RNA structures (GORS) can limit the diversification of RNA viruses, including HVs (Simmonds 2004). GORS can be measured by MFED calculated across a sliding window along the complete genome. The sloth HV showed a low mean MFED (2.5%), 4× lower than HVs found in other mammalian orders outside rodents (Fig. 2A and B) (Simmonds 2004) and 2× lower than in the genetically most related rodent HVs (4.3% in rodent HV MG211815/RHV-GS2015 and 4.5% in rodent HV H-KJ950939/NrHV2). Whether apparently lower GORS in the sloth HV may have aided adaptation to new hosts by lower evolutionary constrains from the maintenance of RNA secondary structures or whether the observed GORS pattern evolved within sloths is an intriguing question.

Further characterization of sloth HV genome termini showed that the 5′-untranslated region (UTR) comprised 427 nt that contained a typical predicted type 4 internal ribosomal entry site (IRES) (Fig. 2C). The sloth HV IRES resembled HCV and other type 4 IRES structures in the characteristic large stem-loop III and the pseudo-knot preceding the initiation codon (Drexler et al. 2013). The 5′ stretch of the IRES showed one microRNA-122 (miR122)-binding site (ACACUCC) with the exact seed sequence of HCV (Jopling 2005) (encased in red (Fig. 2C)). In contrast to HCV, that contains two miR122-binding sites, only one miR122-binding site has been observed in the majority of rodent HVs (Smith et al. 2016). Amongst vertebrates, the miR-122 is conserved and highly expressed in liver tissues, thus suggesting liver tropism of the sloth HV (Jopling 2012). After the stop codon of the polyprotein gene, a 3′-UTR of 123 nt was found, that assembled into three stem-loop structures resembling the structure of the HCV 3′-UTR region termed X-tail (Imbert et al. 2003). A 13 nt A–U-rich region (encased in red (Fig. 2C)) was found. U-rich stretches vary in length between different HV species and even within species, ranging from only three in some rodent HVs to a variable 30–100 nt in HCV (You and Rice 2008; Smith et al. 2016). In sum, the genomic organization of sloth HV showed typical HV characteristics and stark structural similarity to HCV in the viral 3′-genome terminus.

### 3.5 Ancestral state reconstructions

**Figure 3.**
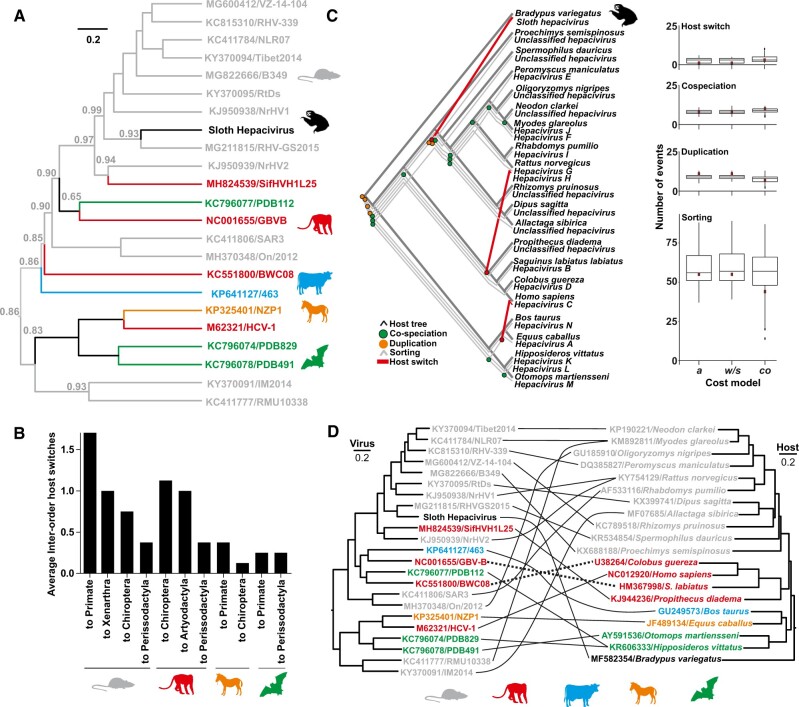
Evolutionary analyses of the sloth hepacivirus. (A) Ancestral state reconstruction (ASR); numbers at nodes denote posterior probability support for a host from the order Rodentia. (B) Summary of inter-order host switches averaged over 15,000 trees from panel 2A. (C) Representation of host and hepacivirus phylogenies. Frequencies of evolutionary events (indicated above panels) in the complete dataset are shown. Tukey box plots show data from hundred randomizations of host–virus associations; *a*, automated cost model; *w*/*s*, automated model excluding reconstructions without host switches; *co*, reconstructions facilitating co-speciations by lower event costs. Black points represent outliers. CoRe-PA quality score: <0.001. (D) Individual host–virus associations tested using ParaFit. Dashed lines denote significant associations P < 0.05. Designations give GenBank accession number and strain names.

To probe sloth HV origins, we conducted ancestral state reconstructions (ASRs) in a Bayesian framework (Suchard et al. 2018) using all mammalian orders and superorders in which unique HVs have been found as traits (Xenarthra, Rodentia, Chiroptera, Primates, Perissodactyla, and Artiodactyla). The most recent common ancestor (MRCA) of the sloth HV was strongly projected to a rodent origin over the aggregated non-rodent host taxa (posterior probability: 0.93; Bayes factor 13.3 (Kass and Raftery 1995)) (Fig. 3A). Notably, the ASR also yielded substantial support for a rodent origin at the root of the HV tree (posterior probability: 0.86; Bayes factor 6.1 (Kass and Raftery 1995)) (Fig. 3A). The importance of rodent hosts during HV evolution was further substantiated by parsimony-based ASRs which showed that rodents are predominant sources of inter-order HV host switches compared to other mammalian HV host taxa (Fig. 3B).

### 3.6 Co-evolutionary analyses

To further assess the evolutionary history of sloth HV, we compared the topologies of host and HV phylogenies and assessed the nature and frequency of host-dependent (co-speciation) and host-independent evolutionary events (sorting, duplication, and host switch) (Merkle et al. 2010). Event-based reconciliations revealed a total of thirteen co-speciations, six duplications (viral, but not host speciation), forty-three sorting events (host, but not viral speciation), and three host switches, namely from rodents to sloths, rodent to lemurs, and from horses to humans (Fig. 3C). Reconciliations that used the same cost model but excluding reconstructions without at least one host switch (*w*/*s*) showed similar results to the automated model, whereas reconciliations that facilitated co-speciation events by lower attributed event costs (*co*) showed a higher number of host switches and less sorting events than the automated model (Fig. 3C, right panel). Host switches were thus consistently predicted irrespective of the model used for co-evolutionary reconstructions. The majority of reconstructed events were sorting events, which is not an uncommon result of event-based reconstructions of viral macro-evolutionary patterns (de Oliveira Carneiro et al. 2018) and that may comprise under-recognized co-speciations of which one counterpart has not been discovered yet or has gone extinct. Co-speciations were reconstructed mostly for rodent (n = 7) and chiropteran (n = 2) nodes and are consistent with prolonged evolutionary associations of HVs with small mammals. However, rodent-associated HVs were also most abundant in the dataset and these results must thus be interpreted with caution. The only co-speciation including primate HVs was observed in a basal node which was subsequently mapped as a host switch into a lemur in apical nodes. In addition, sequence distance-based co-evolutionary reconstructions (Legendre et al. 2002) did not support co-speciation for the whole dataset, and individually significant HV–host relationships were only observed for two HVs detected in the non-human primate species *Colobus guereza* and *Saguinus labiatus* (dashed lines (Fig. 3D)). The limited evidence for co-speciation in primates thus did not support co-evolutionary scenarios to explain the rise of HCV in humans. This was in stark contrast with the evolutionary evidence for partially co-evolutionary relationships observed in another human hepatitis virus, the hepatitis B virus, using comparable methodology (de Carvalho Dominguez Souza et al. 2018). In sum, evolutionary reconciliations strongly suggested a rodent origin of the sloth HV, highlighted the importance of rodents as ancestral hosts of HVs and the ability of HVs to infect genetically divergent hosts.

### 3.7 Conservation of hepaciviral entry

**Figure 4.**
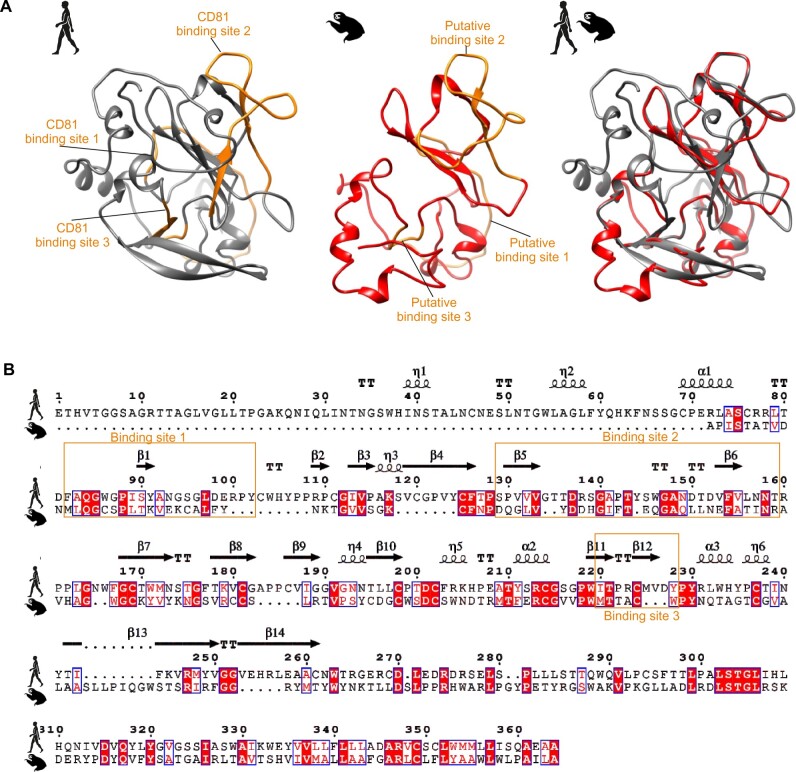
Structural comparison of HCV and sloth HV E2 proteins. (A) Thermodynamic modeling of the E2 of sloth HV (shown in red) and HCV (PDB accession code 4MWF; shown in gray) (Lavie et al. 2015). Known CD81-binding sites for HCV E2 (Lavie et al. 2015) and predicted binding sites for the sloth HV E2 are marked in orange. (B) Conservation of predicted structural elements within the E2 proteins of HCV and sloth HV. Conserved characters are indicated with red boxes; α-helices and 310-helices (η) are represented by squiggles, β-strands are represented by arrows, and strict β-turns are indicated by ‘TT’. Putative CD81-binding sites are encased in orange as in (A).

Cellular entry is a major barrier preventing viral host switches (Drosten 2013). HCV attachment and entry encompass non-specific binding to low-density lipoprotein receptors and glycosaminoglycans, followed by specific binding of three exposed regions of the viral E2 protein to tetraspanin (CD81) (shown in orange (Fig. 4A)), and of the E2 hypervariable region 1 to human scavenger receptor class B type I (SRB1) (Moradpour et al. 2007; Lavie et al. 2015). The tight junction-associated molecules claudin 1 (CLDN1) and occludin (OCLN) are additionally needed as late stage co-receptors for HCV internalization (Pileri 1998; Scarselli et al. 2002; Evans et al. 2007; Ploss et al. 2009). To investigate the viral determinants of sloth HV entry, we modeled the sloth E2 onto structural data available for HCV (Lavie et al. 2015) (Fig. 4A). Despite many differences in primary sequences, there were several structural similarities, including potentially homologous sloth HV E2-binding sites for interaction with CD81 (Lavie et al. 2015) (Fig. 4A and B). These data suggested potentially conserved modes of entry between HCV and sloth HV, that can potentially be extrapolated to other HVs including rodent-borne ancestors of sloth HV.

**Figure 5.**
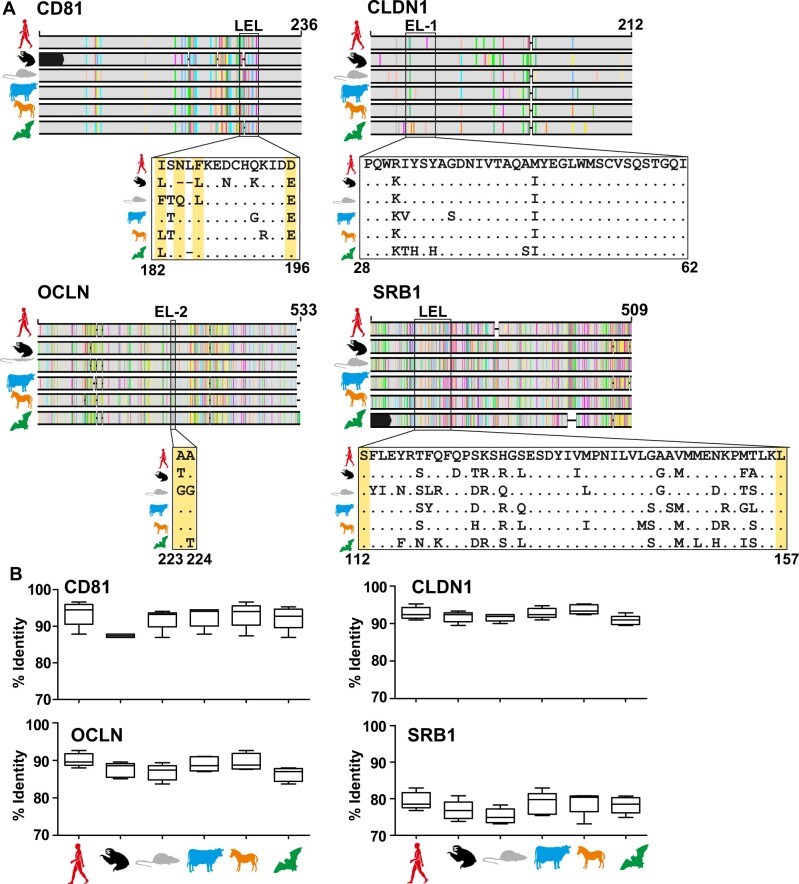
Host restriction factors. (A) Identity of hepacivirus entry molecules across host orders: tetraspanin (CD81), scavenger receptor class B type I (SRB1), tight junction (CLDN1), and occludin (OCLN) for one representative of each order or superorder. Sequences used for the alignment: *C.hoffmanni* scaffold (GenBank number GCA_000164785.2); *R.norvegicus* CD81 (NC_005100), SRB1 (25073), CLDN1 (65129), and OCLN (83497); *Bos taurus* CD81 (511435), SRB1 (282346), CLDN1 (414922), and OCLN (512405); *Equus caballus* CD81 (100060480), SRB1 (100061529), CLDN1 (100059811), and OCLN (100073306); *Homo sapiens* CD81 (975), SRB1 (949), CLDN1 (9076), and OCLN (4950); *Desmodus rotundus* CD81 (NW_020093552), SRB1 (112310844), CLDN1 (112300966), and OCLN (112321073). Missing sequence is shown in black boxes in the alignment sketch above. Boxes below the alignment sketch show the extracellular loop motives of the different molecules associated with HCV entry (Flint et al. 2006; Evans et al. 2007; Michta et al. 2010; Sourisseau et al. 2013; Westhaus et al. 2017). EL, extracellular loop; LEL, large extracellular loop. Critical amino acid residues required for HCV entry are marked with numbers in the *H.sapiens* molecule and shown in yellow background shading (Flint et al. 2006; Evans et al. 2007; Michta et al. 2010; Sourisseau et al. 2013; Westhaus et al. 2017). (B) Box plots of mean distances between orders (line), and minimum and maximum values as whiskers.

Next, to investigate the host determinants of HV entry, we compared HCV entry receptor orthologs. Sloths are very ancient mammals and hypothetically, low homology of cellular receptors may be a major factor limiting HV host switches into sloths, including that projected from rodent-borne ancestors (Evans et al. 2007). The *C.hoffmanni* genome scaffold, albeit without predicted genes, is available in GenBank (GCA_000164785.2). Using the scaffold genome, we were able to recover >90 per cent complete or fully complete coding sequences of HCV entry receptor sloth orthologs (Fig. 5A). The genetic identity of the four molecules associated with HCV entry was variable. The CD81, CLDN1, and OCLN orthologs generally showed above 85 per cent translated sequence identity between mammalian orders, whereas SRB1 orthologs showed slightly lower mutual sequence identity of around 78 per cent (Fig. 5B). However, SRB1 amino acid residues S112 and L157, shown to be critical for HCV binding to human cells were completely conserved across mammals, including sloths (Westhaus et al. 2017). The critical residues of the late-stage co-receptor orthologs (CLDN1 and OCLN) were also relatively conserved across all mammals (Fig. 5A). In contrast, the four CD81 amino acid residues that are critical for HCV entry were more divergent between mammals (highlighted in yellow background, Fig. 5A) (Flint et al. 2006). In addition, the complete sloth CD81 was substantially divergent from other mammalian CD81 orthologs (Fig. 5B). On the one hand, this may suggest that CD81 may be a major barrier against HV host switches, including that projected from rodents into sloths. On the other hand, it may be possible that despite significantly higher sequence distance averaged over the near-complete translated gene, conservation of the critical CD81 residues L186 and E196 between rats and sloths (Fig. 5A) might be compatible with binding of rodent-borne HVs to sloth CD81. In sum, the partial conservation of receptor entry molecule residues interacting with HCV across mammalian orthologs may be compatible with our evolutionary reconstructions projecting an ancestral host switch from rodents into sloths. Nevertheless, in vitro binding and entry experiments will be required to reach definite conclusions on the capacity of HVs to circumvent the entry-associated species barrier.

### 3.8 Selection pressure in rodent entry receptor orthologs

Since our evolutionary reconstructions suggest that rodents are major HV reservoirs, long evolutionary associations may have left signatures of positive selection in entry receptors (Forni et al. 2018). This hypothesis seems at odds with a preliminary study that analyzed selection signatures of CD81, CLDN1, OCLN, and SRB1 in different mammalian orders and found evidence for positive selection only in chiropteran, but not in rodent, CD81 (Forni et al. 2018). Using an updated dataset of twenty-five rodent species of which seventeen species belong to families with at least one known HV host (five more species than in the preliminary analysis, Supplementary Table S2), we found strong evidence of purifying selection in the coding sequences of CD81 orthologs using FUBAR (137 of 236 sites), REL (127 sites), FEL (126 sites), and SLAC (seventy-two sites). This was further substantiated by PamLX, in which the model allowing neutral/purifying selection was a better fit to the data (M8 (beta & ω) vs M7 (beta); log-likelihood scores: 1.2; df: 2; P = 0.5). In contrast to the preliminary study (Forni et al. 2018), we found positive selection in rodent CD81 in two sites (S179 and N180) using FUBAR, and in five partially overlapping sites (N18, F21, N173, S179, I181) in MEME and of gene-wide episodic diversifying selection in BUSTED (P = 0.004) (Supplementary Table S3). Notably, all sites under positive selection except N18 and F21, that were predicted in MEME only and whose robustness is thus less clear, were located in the large extracellular loop of CD81 which interacts with HCV in humans (Forni et al. 2018). Thus, selection pressure signatures in rodent CD81 are consistent with long-term micro-evolutionary patterns in response to interactions with HVs, albeit divergent factors exerting pressure on CD81 orthologs and sampling biases cannot be excluded.

### 3.9 Potential conservation of sloth hepacivirus immune evasion

**Figure 6.**
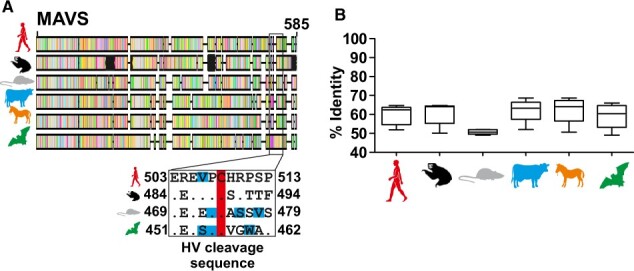
Host immune evasion restriction factors. (A) Mitochondrial antiviral signaling protein (MAVS), alignment. Missing sequence is shown in black boxes in the alignment sketch. Below the genome sketch, the hepacivirus MAVS cleavage sequence alignment is shown. Representative MAVS coding sequences from *Hipposideros armiger* (XM019644596), *H.sapiens* (DQ174270), and *R.norvegicus* (XM_006235035) are shown with the *C.hoffmanni* scaffold-derived predicted sequence. The conserved cysteine residues at the cleavage site are encased in red. Positively selected sites found in the HV cleavage sequence and adjacent residues (Feng et al. 2019) are shown in blue; Primate, V506 using PamL; Rodent, L476, S479, and I481 using REL and PamL; Bat, A484, R485, and E489 using REL and PamL (Feng et al. 2019). (B) MAVS box plots of mean distances between orders (line), and minimum and maximum values as whiskers.

Beyond viral entry, the host’s immune response is a second major barrier preventing viral host switches. Cleavage of Mitochondrial antiviral signaling protein (MAVS) by the HCV NS3/4A is a major determinant of immune evasion and intra-host persistence (Meylan et al. 2005). Strong experimental evidence for heterologous cleavage of human MAVS by equine, bat, rodent, and primate HVs at the canonical HCV cleavage site involving the residues cysteine 508 and histidine 509 (numbered according to the human MAVS coding sequence) (summarized in Rasche et al. (2019)) substantiated that both MAVS cleavage *per se* and the site of cleavage may be conserved HV traits (Anggakusuma et al. 2016). Again, using the *C.hoffmanni* full genome, we were able to retrieve an >90 per cent complete MAVS coding sequence (Fig. 6A). MAVS genetic identity between orders was around 50–65 per cent, which was much lower than the identity observed for entry orthologs and compatible with selection processes associated with an evolutionary arms race between viruses and their hosts (Feng et al. 2019) (Fig. 6B). Distance comparisons showed that only the rodent MAVS was substantially more divergent from other MAVS orthologs (Fig. 6B). Rodent MAVS divergence may thus again be consistent with long-term evolutionary associations with HVs. Irrespective of gene-wide sequence distances that may be shaped by different evolutionary constraints, the MAVS motif determining cleavage by HCV comprises only a short region (^503^EREVPC/H^509^ in human MAVS; Fig. 6A) (Li et al. 2005). Previous studies from us and others analyzed selection pressure signatures in MAVS orthologs of comprehensive primate, rodent, and bat datasets (Patel et al. 2012; Feng et al. 2019). These studies yielded evidence for positively selected sites in the HCV cleavage sequence in these major HV host orders (sites under positive pressure in those studies are highlighted in blue (Fig. 6A)). No different from human MAVS, the critical cysteine residue and the preceding residues upstream of the cleavage site were highly conserved in sloth MAVS (Fig. 6A). Although only hypothetical, MAVS sequence comparisons and selection pressure signatures are thus consistent with long-term evolutionary associations of rodents with HVs. Our data suggest conserved immune evasion of sloth HVs by MAVS cleavage and the ability for predicted rodent-borne ancestral HVs to cleave the sloth MAVS upon the initial host switch. However, our results should be interpreted with caution until verification by functional data.

## 4. Discussion

Here, we characterize a new sloth HV and conduct preliminary investigations into viral infection patterns. Although larger HV datasets will be required to yield definite assertions on HV macro-evolutionary patterns, our data show that rodents are major sources of HV host switches, including that from rodents into sloths. The predicted relevance of rodents for HV origins is consistent the evolutionary origins of numerous zoonotic viruses in rodents, including hanta- and arenaviruses and the likely ancestors of human HAV (Drewes et al. 2017).

The projection of a rodent origin of sloth HV and the detection of genetically closely related HV in two individual sloths requires virus transmission between those animals. Transmission routes of HVs beyond the blood-borne HCV are poorly studied (summarized in Rasche et al. (2019)). Potential routes of transmission between sloths include mating behavior, that is competition for mates or sexual intercourse, or aggressive territorial behavior (Pedrosa et al. 2019), transmission during birth or lactation and finally vector-borne transmission. Both RT-PCR-positive individuals were adults and kept for three and four years in the sanctuary prior to sampling, refuting a recent HV infection in the wild. The low genetic diversity between both HV strains and lack of detectable antibody responses in the two infected animals speaks against vertical transmission. Albeit both sloths were sexually mature adults (Gilmore et al. 2000), they were males kept at separate enclosures, ruling out mating and aggressive behavior as potential modes of horizontal transmission. In turn, aggressive behavior between sloths and rodents during competition for space or resources or by consumption of an infected rodent or rodent material might be a hypothetical transmission route in the wild. Notably, only two-toed sloths are considered omnivorous (Gilmore et al. 2001; Raines 2005), whereas three-toed sloths, that were acutely infected with HV in this study, are fully herbivorous. Accidental consumption of leaves contaminated with rodent material collected in the jungle to feed the captive sloths could thus be an additional plausible transmission route.

Albeit vector-borne transmission of HCV was hypothesized based on mathematical models of endemic HCV transmission patterns (Pybus et al. 2007), neither HCV nor any other HV is known to be transmitted through invertebrate vectors. However, rodent-associated blood sucking *Polygenis atopus* fleas that are occasionally found in sloths cannot be ruled out as an hypothetical transmission route between rodents and sloths (Tipton and Machado-Allison 1972).

Finally, iatrogenic transmission may be possible between sloths, as the only possible contact between the two sloths would be indirect through routine handling by veterinarians or caretakers. Unfortunately, no data on prior veterinary procedures were available to infer potential iatrogenic transmission and no prospective samples were available to test if the animals remained RT-PCR-positive over prolonged periods.

Our results also imply that an origin of HCV as a result of a non-recent host switch from an unknown source seems plausible (Pybus and Gray 2013). The quest for HCV ancestors should thus focus on rodents and other small mammals. Notably, this hypothesis is not contradictory to the relatively higher genetic relatedness of equine HVs with HCV compared to other HVs, since equine HVs may have recent evolutionary origins in equids because of their limited genetic diversity (Walter et al. 2017). Hypothetically, equine HVs themselves thus correspond to another host switch from an unknown source, potentially including rodents.

The HV genealogy includes striking genetic relatedness between viruses found in divergent hosts and at extreme geographical separation, exemplified by the genetic relatedness of the Costa Rican sloth HV and a Chinese rodent HV. It will be intriguing to investigate whether the genetic relatedness across large distances is due to non-recent HV spread by ancestral hosts followed by local host switches, or whether there may be contemporary intermediate hosts. Albeit highly speculative, the relevance of rodents for the HV genealogy suggested by our data may hint at Norway or Brown rats, the only globally occurring rodents, as important bridging hosts. This hypothesis is consistent with the ability to experimentally adapt Norway rat HV-1 to mice (Billerbeck et al. 2017), with the relatively high genetic diversity of HVs in Norway rats in a single location (Firth et al. 2014) and with the role of rats to spread other viruses, such as morbillivirus-related viruses between bats and other small mammals in an island ecosystem (Wilkinson et al. 2014). However, rodents other than rats, such as bank voles, also harbor genetically highly divergent HVs (Drexler et al. 2013). In addition, other intermediate hosts like bats might exist and spread HVs across relatively large geographical distances, facilitated by their ability to fly and particular ecological traits (Lukashev et al. 2017; Sander et al. 2018). Whether rodents are indeed more important for HV spread and evolution than bats thus remains an open question. Notably, studies analyzing thousands of bats and rodents have yielded relatively higher HV genetic diversity and detection rates in rodents than in bats (Drexler et al. 2013; Quan et al. 2013). However, a sampling or technical bias cannot be ruled out and large comparative studies assessing the diversity of HVs in rodents and bats sampled across large geographic distances will be needed.

In sum, rodents are major hosts driving spread of HVs between genetically divergent hosts and across large geographic distances. It seems plausible that host switches have occurred during the HV evolutionary history to an under-recognized extent, likely including the origins of human HCV.

## Data availability

Viral genomic data is available in GenBank under accession numbers MH844500 and MH844501. Data on selection analyses are available in the Supplementary Material. All the other data are available upon request.

## Supplementary data

Supplementary data are available at *Virus Evolution* online.

## Supplementary Material

veaa033_Supplementary_Data
