# Supplementary material for: Cross-order host switches of hepatitis C-related viruses illustrated by a novel hepacivirus from sloths
Source: Virus Evol. 2020 Apr 25;6(2):veaa033. doi: 10.1093/ve/veaa033 (PMC7368370; doi:10.1093/ve/veaa033)
Supplement: veaa033_Supplementary_Data [file ve_6_2_veaa033_s7.docx]

**Supplementary Table 1. Oligonucleotides used for the quantification and amplification of the sloth hepacivirus genome**

| **Name** | **Sequence** |
| --- | --- |
| 3utr slothhv R8853 | CCAACCACCACTGGAACG |
| 3utr slothhv R8831 | GAGCCGAGACGGTTCTTCC |
| slothhv AS8 R8801 | GTCACACCCTCTACCTTGCC |
| slothhv i AS7 R7952 | GCACACACAATCGTCACCAC |
| slothhv AS7nest R7917 | ACCACAAATCAACCATTCCG |
| slothhv AS8 nest F7745 | GGATTCGCACTCTCCATAG |
| slothhv AS8 F7580 | CCAAGAACGGGTGGGTAGAC |
| slothhv AS6 R6854 | GAAACTAGCACATTAGGATCAGC |
| slothhv AS6 nest R6440 | TTGCCCACATCTCTTCCACC |
| slothhv AS7 nest F6236 | AGCCTCTTGGAACACCATGG |
| slothhv AS7 F5971 | GGTTGGTGGTCGAATTTGGC |
| slothhv AS5 R5308 | CTGTATCCCTGCTAAGATGTGTGG |
| slothhv AS6 F5029 | AGCAGGGTTAGGGCTAGCAG |
| slothhv AS4 R4048 | GACTTAAGAGCACTACCTATCC |
| slothhv AS4 nest R3977 | GACATCGCACTCTAATTGAGAACC |
| slothhv AS5 nest F3885 | TACGGATTCACACCCAGCAC |
| slothhv AS5 F3751 | AGATAGTTGCCCCTACCGGT |
| slothhv AS3 R2838 | CCGTACTGCCTTGTATAAGCC |
| slothhv AS3 nest R2792 | CTTCCACACAAATTAACCCACAC |
| slothhv AS 4 nest F2073 | GAATCTATGTTCTTATGCATCC |
| slothhv AS 4 F2633 | ATGCTAGTGTTCATGTTCCATGG |
| slothhv AS2 R1776 | CCAACCAGCATGGACTGCTC |
| slothhv AS3 nest R1671 | GTGTCGGGCAAATGTTTCAATCC |
| slothhv AS3 F1621 | GGAGAGCAACCCTGAAGCAT |
| slothhv AS1 R728 | CGTTTACATGCAGCGGATCG |
| slothhv AS1 R687 | CGAGATGCTAGGTGGCGTAG |
| slothhv AS2 F512 | GTCTTGCCGCAAGGGTTCAGG |
| slothhv AS2 F495 | GCTGACACTGCCTGATAGG |
| slothhv AS1 F228 utr | CCACAACGTCAGGTCTGTCG |
| 5utrslothhv F82 | ACCTCCCTGGCACAGTGTAC |
| 5utrslothhv F32 | TCTGTGGGACATGTCCTTGG |
| *SlothHVNS5B-rtF | CGGGCTCTACCATTACATGA |
| *SlothHVNS5B-rtP | TAGAGATCCTGCAATTCCGCTGGCC |
| *SlothHVNS5B-rtR | CCGTTTTCCCTCAAGTGATGA |

* Real time RT-PCR primers and probe (5’ labelled with FAM, 3’ labelled with a dark quencher)

**Supplementary Table 2. Rodent species used for CD81 selection pressure analyses**

| **Scientific name *(known HV host)** | **Common name** | **Rodent Family *(at least one HV host inside the family)** | **Genbank ID** |
| --- | --- | --- | --- |
| *Rattus norvegicus** | Norway rat | Muridae* | NP_037219.2 |
| *Mus musculus* | House mouse | Muridae* | NP_598416.1 |
| *Cavia porcellus* | Domestic guinea pig | Caviidae | XP_003468385.1 |
| *Cricetulus griseus* | Chinese hamster | Cricetidae* | XP_003508125.1 |
| *Octodon degu* | Degu | Octodontidae | XP_004627145.1 |
| *Jaculus jaculus* | Lesser Egyptian jerboa | Dipodidae* | XP_004654280.1 |
| *Heterocephalus glaber* | Naked mole-rat | Heterocephalidae | XP_004852159.1 |
| *Mesocricetus auratus* | Golden hamster | Cricetidae* | XP_005064201.1 |
| *Microtus ochrogaster* | Prairie vole | Cricetidae* | XP_005351632.1 |
| *Chinchilla lanigera* | Long-tailed chinchilla | Chinchillidae | XP_005384261.1 |
| *Peromyscus maniculatus bairdii* | Prairie deer mouse | Cricetidae* | XP_006977357.1 |
| *Spalax galili* | Upper Galilee mountains blind mole rat | Spalacidae* | XP_008837422.1 |
| *Fukomys damarensis* | Damara mole rat | Bathyergidae | XP_010633959.1 |
| *Ochotona princeps* | American pika | Ochotonidae | XP_012786434.1 |
| *Dipodomys ordii* | Ords kangaroo rat | Heteromyidae | XP_012876643.1 |
| *Marmota marmota* | Alpine marmot | Sciuridae* | XP_015358813.1 |
| *Castor canadensis* | American beaver | Castoridae | XP_020024374.1 |
| *Mus caroli* | Ryukyu mouse | Muridae* | XP_021022691.1 |
| *Pseudohydromys musseri* | Shrew mouse | Muridae* | XP_021056319.1 |
| *Meriones unguiculatus* | Mongolian gerbil | Muridae* | XP_021489403.1 |
| *Ictidomys tridecemlineatus* | Thirteen-lined ground squirrel | Sciuridae* | XP_021575956.1 |
| *Urocitellus parryii* | Arctic ground squirrel | Sciuridae* | XP_026267091.1 |
| *Marmota flaviventris* | Yellow-bellied marmot | Sciuridae* | XP_027800533.1 |
| *Grammomys dolichurus* | African woodland thicket rat | Muridae* | XP_028627087.1 |
| *Peromyscus leucopus* | White-footed mouse | Cricetidae* | XP_028726722.1 |

**Supplementary Table 3. Evidence for positive selection in rodent CD81 orthologs**

| **AA^1^** | **SLAC** | **FEL** | **REL** | **FUBAR** | **MEME** | **PamLX** |
| --- | --- | --- | --- | --- | --- | --- |
| N18 | n.s. | n.s. | n.s. | n.s. | 0.00 | n.s. |
| F21 | n.s. | n.s. | n.s. | n.s. | 0.04 | n.s. |
| **N173** | n.s. | n.s. | n.s. | n.s. | 0.08 | n.s. |
| **S179** | n.s. | n.s. | n.s. | 0.923 | 0.06 | n.s. |
| **N180** | n.s. | n.s. | n.s. | 0.922 | n.s. | n.s. |
| **I181** | n.s. | n.s. | n.s. | n.s. | 0.1 | n.s. |

Statistical criteria: SLAC, FEL, MEME p≤0.1, REL Bayes Factor >50, FUBAR and M8 BEB PPr>0.9. n.s., not significant

^1^AA position in Arctic ground squirrel CD81

Bold, residues within the CD81 large extracellular loop that interacts with HCV
